# Longitudinal microcomputed tomography detects onset and progression of pulmonary fibrosis in conditional *Nedd4-2* deficient mice

**DOI:** 10.1152/ajplung.00280.2023

**Published:** 2024-10-22

**Authors:** Dominik H. W. Leitz, Philip Konietzke, Willi L. Wagner, Mara Mertiny, Claudia Benke, Thomas Schneider, Rory E. Morty, Christian Dullin, Wolfram Stiller, Hans-Ulrich Kauczor, Marcus A. Mall, Julia Duerr, Mark O. Wielpütz

**Affiliations:** ^1^Department of Pediatric Respiratory Medicine, Immunology and Intensive Care Medicine, Charité – Universitätsmedizin Berlin, Berlin, Germany; ^2^German Center for Lung Research (DZL), Berlin, Germany; ^3^Berlin Institute of Health (BIH) at Charité – Universitätsmedizin Berlin, Berlin, Germany; ^4^Translational Lung Research Center Heidelberg (TLRC), German Center for Lung Research (DZL), Heidelberg, Germany; ^5^Department of Diagnostic and Interventional Radiology, University Hospital Heidelberg, Heidelberg, Germany; ^6^Department of Diagnostic and Interventional Radiology with Nuclear Medicine, Thoraxklinik at University Hospital Heidelberg, Heidelberg, Germany; ^7^Department of Translational Pulmonology and the Translational Lung Research Center Heidelberg, University Hospital Heidelberg, member of the German Center for Lung Research (DZL), Heidelberg, Germany; ^8^Translational Molecular Imaging, Max-Plank-Institute for Multidisciplinary Sciences, Göttingen, Germany; ^9^Institute for Diagnostic and Interventional Radiology, University Medical Center, Göttingen, Germany

**Keywords:** animal model, idiopathic pulmonary fibrosis, IPF, micro-CT, Nedd4-2

## Abstract

Idiopathic pulmonary fibrosis (IPF) is a fatal lung disease, which is usually diagnosed late in advanced stages. Little is known about the subclinical development of IPF. We previously generated a mouse model with conditional *Nedd4-2* deficiency (*Nedd4-2^−/−^*) that develops IPF-like lung disease. The aim of this study was to characterize the onset and progression of IPF-like lung disease in conditional *Nedd4-2^−/−^* mice by longitudinal micro-computed tomography (CT). In vivo micro-CT was performed longitudinally in control and conditional *Nedd4-2^−/−^* mice at 1, 2, 3, 4, and 5 mo after doxycycline induction. Furthermore, terminal in vivo micro-CT followed by pulmonary function testing and post mortem micro-CT was performed in age-matched mice. Micro-CT images were evaluated for pulmonary fibrosis using an adapted fibrosis scoring system. Histological assessment of lung collagen content was conducted as well. Micro-CT is sensitive to detect the onset and progression of pulmonary fibrosis in vivo and to quantify distinct radiological IPF-like features along disease development in conditional *Nedd4-2^−/−^* mice. Nonspecific interstitial alterations were detected from 3 mo, whereas key features such as honeycombing-like lesions were detected from 4 mo onward. Pulmonary function correlated well with in vivo (*r* = −0.738) and post mortem (*r* = −0.633) micro-CT fibrosis scores and collagen content. Longitudinal micro-CT enables in vivo monitoring of the onset and progression and detects radiological key features of IPF-like lung disease in conditional *Nedd4-2^−/−^* mice. Our data support micro-CT as a sensitive quantitative endpoint for the preclinical evaluation of novel antifibrotic strategies.

**NEW & NOTEWORTHY** IPF diagnosis, particularly in early stages, remains challenging. In this study, micro-CT is used in conditional *Nedd4-2^−/−^* mice to closely monitor the onset and progression of progressive pulmonary fibrosis in vivo. Together with high-resolution post mortem micro-CT, this allowed us to track how nonspecific lung lesions develop into key IPF-like features. This approach offers a noninvasive method to monitor pulmonary fibrosis, providing a quantitative endpoint for the preclinical evaluation of novel antifibrotic strategies.

## Graphical abstract

**Figure:**
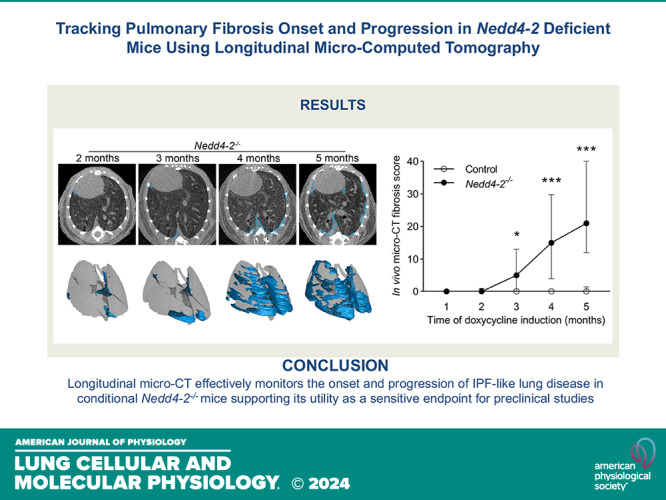

## INTRODUCTION

Idiopathic pulmonary fibrosis (IPF) is a fatal chronic progressive lung disease, which is characterized by aberrant fibrotic remodeling of the peripheral lung. The resulting progressive loss of lung function combined with limited therapeutic options, ultimately leads to respiratory failure (1–5). The diagnosis of IPF is based on the presence of the usual interstitial pneumonia (UIP) pattern in computed tomography (CT) together with the exclusion of known causes of interstitial lung disease using a multidisciplinary diagnostic approach (1). The UIP pattern is characterized by reticular opacities, traction bronchiectasis, and subpleural clusters of cystic airspaces described as “honeycombing,” predominantly in the basal and peripheral areas of the lung, which may have varying temporal patterns of occurrence and progression (1, 6). At clinical diagnosis following the onset of symptoms, irreversible lung damage is already present in most patients (7, 8). Therefore, little is known about the onset of IPF, and early structural changes have therefore not been defined.

Several mouse models of pulmonary fibrosis exist, including bleomycin-induced lung fibrosis (BILF), radiation-induced fibrosis, lung-specific transgenic mice, and models using adenoviral vectors for gene overexpression (9–11). These models have the limitation that pulmonary fibrosis is either transient as the BILF model, or restricted to certain aspects of fibrosis as is the case with transgenic models with the overexpression of endogenous fibrogenic mediators or harboring gene mutations associated with familial forms of IPF, and animal models of progressive pulmonary fibrosis were lacking (12–15). We and others recently found that the expression of *NEDD4-2* is downregulated in IPF (16–19). Nedd4-2 is an E3 ubiquitin protein ligase involved in posttranscriptional regulation by ubiquitination and targeting for proteasomal degradation of proteins implicated in the pathogenesis of lung disease, including surfactant protein C (*Sftpc*), the epithelial sodium channel ENaC, and Smad2/3, the intracellular mediators of transforming growth factor β (TGFβ) signaling (17, 20–23). By conditional deletion of *Nedd4-2* (conditional *Nedd4-2^−/−^*), we previously generated a mouse model that develops spontaneous, chronically progressive lung disease that recapitulates key features of IPF in patients such as a distinct fibrotic pattern in histology and micro-computed tomography (micro-CT) including honeycombing-like lesions and fibroblast foci-like fibrotic consolidations, resulting in restrictive lung disease in pulmonary function testing and high pulmonary mortality from 3 mo after induction onward (17). In our previous work, the characterization of the pulmonary phenotype of conditional *Nedd4-2^−/−^* mice was limited to advanced stages of lung disease 4 mo after doxycycline induction including post mortem micro-CT at ∼15 µm resolution using a modified scoring system derived from the human situation (17, 24, 25).

The aim of this study was to use in vivo micro-CT imaging to longitudinally assess the onset and progression of IPF-like lung disease in spontaneously breathing conditional *Nedd4-2*^−/−^ mice compared with littermate controls. In vivo was complemented by post mortem imaging in age-matched conditional *Nedd4-2*^−/−^ and control mice. The previously developed dedicated micro-CT fibrosis scoring system (17) was used for semiquantitative evaluation of key IPF-like features, such as honeycombing-like lesions, reticulations, and traction bronchiectasis. In addition, we performed pulmonary function testing and histological measurement of lung collagen content to assess the relationship between abnormalities in lung structure and function during the development of IPF-like lung disease in this mouse model. Finally, we assessed the longitudinally scanned mice for radiation-induced lung injury to determine the suitability of micro-CT imaging for in vivo monitoring of disease development and response to novel antifibrotic treatment strategies.

## MATERIALS AND METHODS

### Experimental Animals

All animal studies were approved by the responsible animal protection authority (Project Identification No. 35–9185.81/G-45/14, Regierungspräsidium Karlsruhe, Karlsruhe, Germany). Mice for conditional deletion of *Nedd4-2* in lung epithelial cells were generated as previously described (17). In brief, mice carrying *Nedd4-2^fl/fl^* (26) were intercrossed with *CCSP-rtTA2S-M2* line 38 (*CCSP-rtTA2S-M2*) (27) and LC1 mice (28). All three lines were on a C57BL6/N background. *Nedd4-2^fl/fl^* littermates that showed no abnormalities in prior studies served as controls (17). Mice were housed in a specific pathogen-free animal facility and had free access to food and water. For induction of the *Nedd4-2* deletion, mice were exposed to 1 mg/mL doxycycline hydrochloride (Sigma-Aldrich, Darmstadt, Germany) dissolved in a 5% sucrose solution supplied as drinking water in light-protected bottles. Doxycycline solutions were prepared freshly and changed at least every 3 days. To longitudinally study the lung phenotype, 4–6 wk old mice were continuously treated with doxycycline solution until clinically symptomatic or for exact periods of 1, 2, 3, 4, and 5 mo as indicated. Corresponding survival curves are shown in Supplemental Fig. S3. All mice were included in our study, irrespective of sex, yielding a balanced sex distribution in the conditional *Nedd4-2^−/−^* groups and littermate controls (Supplemental Table S1).

### Pulmonary Function Testing

Mice were anesthetized using sodium pentobarbital (80 mg/kg), tracheotomized, and placed on the FlexiVent system (SCIREQ, Montreal, QC, Canada). After relaxation with pancuronium bromide (0.5 mg/kg), mice were ventilated with a tidal volume of 8 mL/kg at a frequency of 150 breaths/min and a positive end-expiratory pressure of 3 cmH_2_O. The static compliance was derived from pressure-volume curves as described previously (17, 29–34). Mice were then euthanized for post mortem micro-CT.

### Microcomputed Tomography

In vivo longitudinal micro-CT was performed in conditional *Nedd4-2^−/−^* mice and littermate controls at 1, 2, 3, 4, and 5 mo after induction in the supine position using a small animal micro-CT SkyScan 1176, software v. 1.1 (Bruker, Kontich, Belgium). Mice were sedated with 2% isoflurane/2l O_2_ flow inhalation anesthesia via a nasal cone. Scans were acquired in free-breathing with following parameters: 50 kVp X-ray source voltage, 500 µA current, a composite X-ray filter of 0.5 mm aluminum, 55 ms camera exposure time per projection, 720 projections per view, 30 × 30 mm^2^ field of view, acquiring projections with 0.5° increments over a total angle of 360°, resulting in an isotropic pixel size of 35 µm with 1,000 × 1,000 pixel in plane. The scanning time was ∼12 min. During image acquisition, the respiratory movements of the thorax were recorded with a visual camera, and this information was converted into a pseudosinusoidal signal, allowing retrospective respiratory gating. The duration of a complete respiratory cycle was divided into four phases of equal length, from the first inspiration to the late expiration. All images were assigned to the corresponding respiratory phase.

After in vivo imaging and pulmonary function testing, post mortem micro-CT at higher resolution was performed at each timepoint. Mice were euthanized and again placed in the micro-CT. A fixed pressure to 25 cm H_2_O was applied via the tracheal cannula to ensure sufficient inflation of the lung. Scans were acquired with the following parameters: 50 kVp X-ray source voltage, 550 µA current, a composite X-ray filter of 0.5 mm aluminum, 902 ms camera exposure time per projection, 1,440 projections per view, 21 × 31 mm field of view, acquiring projections with 0.25° increments over a total angle of 360°, resulting in an isotropic pixel size of 9 µm with 4,000 × 4.000 pixel in plane. Total scanning time was ∼124 min.

Tomograms were reconstructed using NRecon software v. 1.7.0.4 (Bruker, Kontich, Belgium). The reconstruction parameters were smoothing “2” and beam-hardening correction “44%”; postalignment and ring artifact corrections were manually optimized for each individual scan. For three-dimensional (3-D) visualization of the distribution of fibrotic lesions, the image datasets were processed using VGSTUDIO MAX software v. 3.4.3 (Volume Graphics GmbH, Heidelberg, Germany). Surface-rendering was computed based on differences in density.

### Micro-CT Fibrosis Scoring System

Micro-CT was evaluated for pulmonary fibrosis using an adapted fibrosis scoring system, as described previously (17, 24, 25) with CTAn software v. 1.10.0.0 (SkyScan, Kontich, Belgium) by two independent radiologists (P.K. and T.S.) with 6 and 4 yr of experience in thoracic and small animal imaging, respectively. Consensus was achieved by averaging scores from both readers. In brief, lungs were subdivided into 10 equally spaced sections along the longitudinal *z*-axis, from the thoracic inlet to the dome of diaphragm. At each level, lungs were separated into right and left lung according to the perpendicular line that passes the midline of the sternum. For post mortem and in vivo micro-CT, the scoring system was modified to adapt to the different resolution: The post mortem fibrosis score comprised subscores for consolidations, reticular opacities, peripheral bronchiectasis, honeycombing, parenchymal lines, and fissural thickening. Consolidation defined as an increased attenuation with obscuration of the pulmonary vascular markings, and reticular opacities defined as a linear interstitial opacity and thickening of the peripheral connective tissue septa, were scored on a scale from 0 to 5 depending on the lung area involved: 0: absent; 1: >0% to 10%, 2: >10% to 20%, 3: >20% to 40%, 4: >40% to 60%, and 5: >60%. Honeycombing-like lesions defined the presence of small cystic airspaces with irregularly thickened walls adjacent to the pleural surface, and peripheral bronchiectasis defined as bronchial dilatations with a maximal distance of 1 mm to the pleura were scored on a scale from 0 to 3 depending on the pleural surface involved: 0: absent, 1: >0% to 10%, 2: >10% to 20%, and 3: >20%. Parenchymal lines defined as nonvascular linear structures arising from the pleural surface and fissural thickening were scored on a scale from 0 to 1: 0: absent and 1: present. The maximum score for 9 µm resolution images was [(10×(2 × 5) + 10×(2 × 3) + 10×(2 × 1)] = 180 for each side and 360 for the total lung.

In vivo micro-CT was scored for the area of consolidations as aforementioned for the post mortem fibrosis score, as well as for the number of consolidations from 0 (absent) to 5, resulting in a maximum score of 10× (2 × 5) = 100 for both the right or the left lung, and a maximum score of 200 for the total lung.

### Interobserver Reliability

To establish a micro-CT-based scoring system for standardized quantification of pulmonary fibrosis with focus on IPF characteristics in mice, we modified a fibrosis score already used in previous publications (24, 25) and adapted it for use in in vivo and post mortem micro-CT. All images were scored independently by two experienced thoracic radiologists, and the weighted Kohens kappa (κ) was calculated to evaluate interrater reliability (Supplemental Table S2). For in vivo micro-CT, almost perfect agreement was achieved for the numbers and areas of consolidations (κ = 0.83) and a substantial agreement for the in vivo total micro-CT fibrosis score (κ = 0.79). For post mortem micro-CT, almost perfect interobserver agreement was achieved for the consolidations (κ = 0.96), fissural thickening (κ = 0.86), and honeycombing-like (κ = 0.84) subscores. Substantial agreement was met for the peripheral bronchiectasis (κ = 0.76), pleural lines (κ = 0.65), and reticulations (κ = 0.63) subscore. The agreement for the total post mortem micro-CT fibrosis score was fair (κ = 0.40) (Supplemental Table S2).

### Radiometry

The radiation dose was estimated using a LiF: Mg/Cu/P-thermoluminescent dosimeter (TLD) also called GR-200A (Solid Dosimetric Detector & Method Laboratory, Beijing, China) (35), individually calibrated with respect to a CS 137 radioactive source of known activity. The TLD was scanned five times using the in vivo protocol, and the registered dose was averaged. The cumulative equivalent dose measured was 1,983 mSv in aqua after five scans, resulting in 393 mSv as an approximation to the dose applied per scan in mice.

### Histology

Lungs were fixed in 4% buffered formalin. After paraffin embedding, lungs were processed for histology, sectioned at 5 μm, and stained with hematoxylin and eosin (H&E) and picrosirius red staining. Histological images were captured with a NanoZoomer S60 Slidescanner (Hamamatsu Photonics K.K., Hamamatsu, Japan) using NDP.view2 software v. 2.8.24 (Hamamatsu Photonics K.K., Hamamatsu, Japan).

### Collagen Quantification

Collagen content in lung parenchyma was quantified in picrosirius red-stained lung sections using the open-source image analysis software, ImageJ (v. 1.49, National Institutes of Health). In each lung section, 10 regions of interest (ROI) with a size of 750 µm × 750 µm, located in the lung parenchyma without big vessels or airways were selected. Red-stained collagen fibers were segmented using the color threshold tool. The collagen area fraction of each ROI was calculated by dividing the area of pixels representing collagen-stained fibers by the area of the ROI, and the median collagen area fraction was calculated for each mouse (36).

### Statistical Analyses

All data are shown as median ± interquartile range. Data were analyzed with GraphPad Prism v. 9 (GraphPad Software Inc, LaJolla, CA). For comparison of two groups, Mann–Whitney *U* test was used. No outliers were excluded from the analyses. Comparison of more than two groups was performed with Kruskal–Wallis test followed by Dunn’s post hoc test. For multiple comparisons, *P* values were adjusted according to the Bonferroni method or by using the false discovery rate (FDR) method proposed by Benjamini et al. (37, 38) as appropriate. The Spearman rank order correlation coefficient (*r*) was calculated for micro-CT fibrosis scores and the pulmonary compliance. We used IBM SPSS Statistics v. 27.0.1.0 (IBM Corp, Armonk, NY) to calculate the weighted Cohen’s kappa (κ) with a squared weight matrix, providing a more accurate assessment of interrater reliability for ordinal data with multiple categories. Survival was compared using the log-rank test, and *P* values were corrected for multiple comparison by the Bonferroni method.

## RESULTS

### Longitudinal In Vivo Micro-CT Imaging Detects the Onset and Progression of Pulmonary Fibrosis in Conditional *Nedd4-2^−/−^* Mice

**Figure 1.**
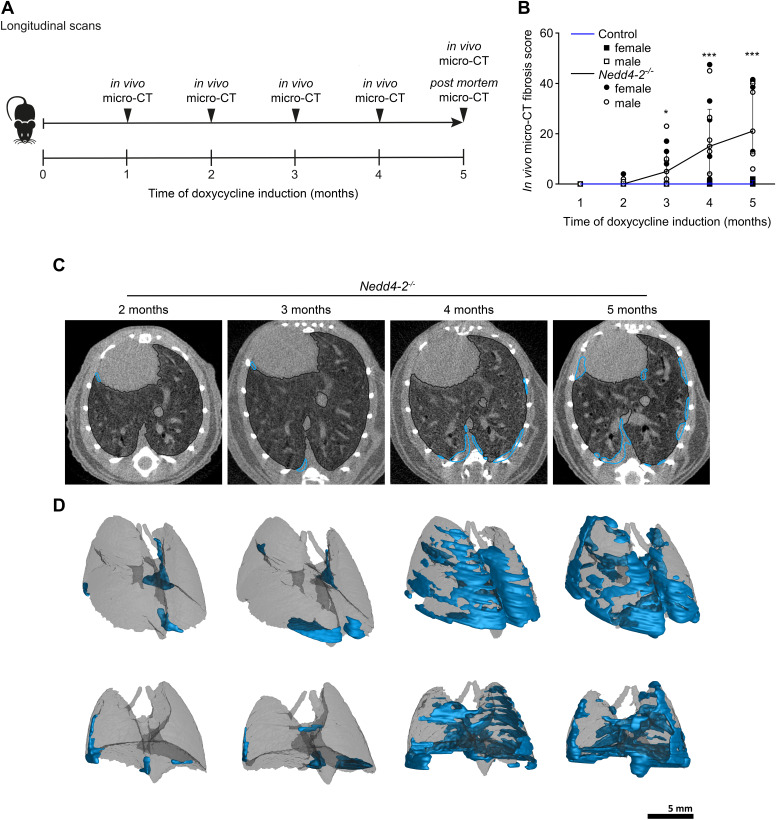
Longitudinal in vivo micro-CT detects onset and progression of pulmonary fibrosis in conditional *Nedd4-2^−/−^* mice. *A*: schematic illustration of the longitudinal study design with serial in vivo micro-CT 1, 2, 3, 4, and 5 mo after doxycycline induction of conditional *Nedd4-2^−/−^* mice and controls. *B*: summary of in vivo micro-CT fibrosis scores assessed by longitudinal micro-CT. *C*: representative longitudinal in vivo micro-CT image of a spontaneously breathing conditional *Nedd4-2^−/−^* mouse scanned at the indicated time points after doxycycline induction. Fibrotic areas are indicated in blue. *D*: 3-D reconstructions of fibrotic lesions (blue) in the lung of the same mouse as shown in *C* after 2, 3, 4, and 5 mo of doxycycline induction. *n* = 5–15 mice/group. *P < 0.05, ***P < 0.001. 3-D, three-dimensional; micro-CT, micro-computed tomography.

To determine the onset and progression of IPF-like lung disease, we performed longitudinal studies using monthly in vivo micro-CT in a cohort of conditional *Nedd4-2^−/−^* mice and controls (Fig. 1; Supplemental Fig. S1). In this study group, further referred to as the longitudinal study group, we performed micro-CT repeatedly in the same mice starting 1 mo after induction for up to 5 mo. Immediately after the fifth scan, we performed pulmonary function testing, and mice were euthanized for a final post mortem scan (Fig. 1*A*). Standardized scoring showed that the fibrosis score was significantly increased at 3 mo after doxycycline induction in conditional *Nedd4-2^−/−^* mice compared with control mice (Fig. 1, *B* and *C*). Micro-CT at 4 and 5 mo showed progressive pulmonary fibrosis with continuously increasing fibrosis scores in conditional *Nedd4-2^−/−^* mice (Fig. 1, *B* and *C*). Sex-stratified subgroup analyses of this study group did not show significant differences between female and male mice (Fig. 1*B*). 3-D reconstructions revealed the spatial distribution of fibrotic lesions in the lungs, which originated in basolateral regions, increased both in number and volume, and extended apically overtime. Distinct fibrotic lesions partially merged into massive fibrotic conglomerates (Fig. 1*D*).

### Comparison of In Vivo and Post Mortem Micro-CT during the Development of IPF-like Lung Disease in Conditional *Nedd4-2^−/−^* Mice

**Figure 2.**
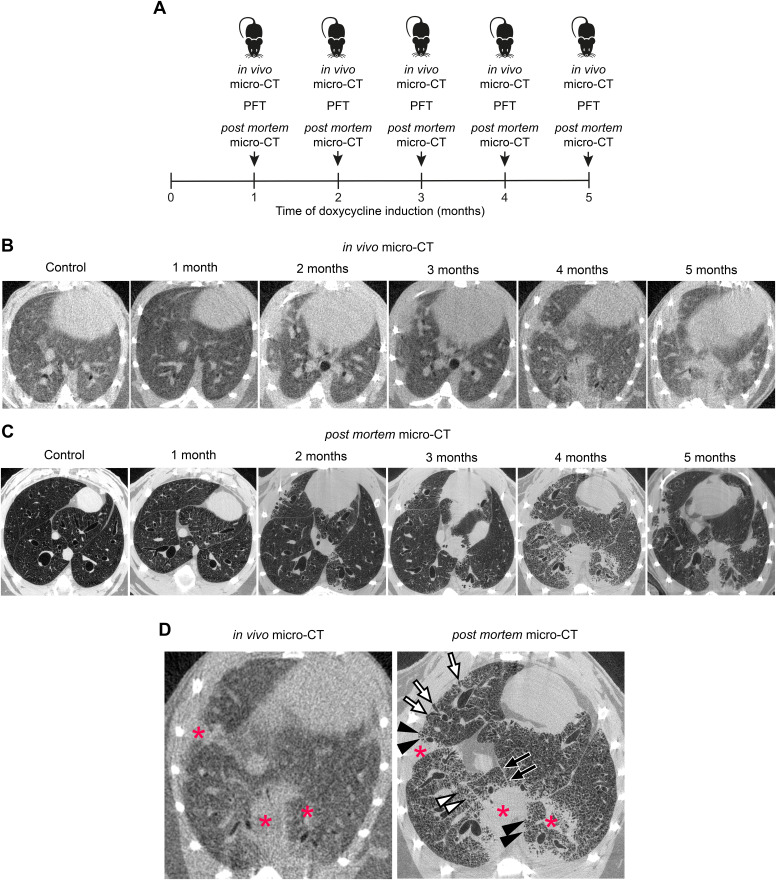
Comparison of radiological features determined by in vivo and post mortem micro-CT during the development of IPF-like lung disease in conditional *Nedd4-2^−/−^* mice. *A*: study design of cross-sectional micro-CT imaging during the development of IPF-like lung disease in conditional *Nedd4-2^−/−^* mice using in vivo micro-CT imaging followed by pulmonary function testing (PFT) and a post mortem micro-CT scan after 1, 2, 3, 4, and 5 mo of doxycycline induction. *B* and *C*: representative in vivo micro-CT images in spontaneously breathing conditional *Nedd4-2^−/−^* mice (*B*) and post mortem micro-CT (*C*) at indicated time points. *D:* Comparison of consolidations (red asterisks), honeycombing-like lesions (black arrowheads), peripheral bronchiectasis (white arrows), parenchymal lines (black arrows), and reticulations (white arrowheads) in in vivo and post mortem micro-CT 4 mo after conditional *Nedd4-2* deletion. IPF, idiopathic pulmonary fibrosis; micro-CT, micro-computed tomography.

To study the onset and progression of IPF-like lung disease in conditional *Nedd4-2^−/−^* mice at higher resolution, we performed in vivo and post mortem micro-CT imaging of conditional *Nedd4-2^−/−^* mice at the same time points as for the longitudinal in vivo studies in a cross-sectional study design (*n* = 5–12 mice/time point) (Fig. 2*A*). In this group, further referred to as the cross-sectional study group, we investigated independent groups after 1, 2, 3, 4, and 5 mo of induction and performed in vivo micro-CT, followed by pulmonary function testing and final post mortem micro-CT imaging.

Similar to in vivo scans, post mortem micro-CT imaging detected initial consolidations after 3 mo of induction in conditional *Nedd4-2^−/−^* mice, but not in littermate controls (Fig. 2, *B* and *C*). In addition, characteristic IPF-like structural alterations of the lung such as honeycombing-like lesions, fissural thickening, peripheral bronchiectasis, parenchymal lines, and reticulations were distinguishable on post mortem, but not on in vivo micro-CT images (Fig. 2*D*).

### Higher Resolution Post Mortem Micro-CT Detects the Onset and Progression of Specific Fibrotic Lesions in IPF-Like Lung Disease in Conditional *Nedd4-2^−/−^* Mice

**Figure 3.**
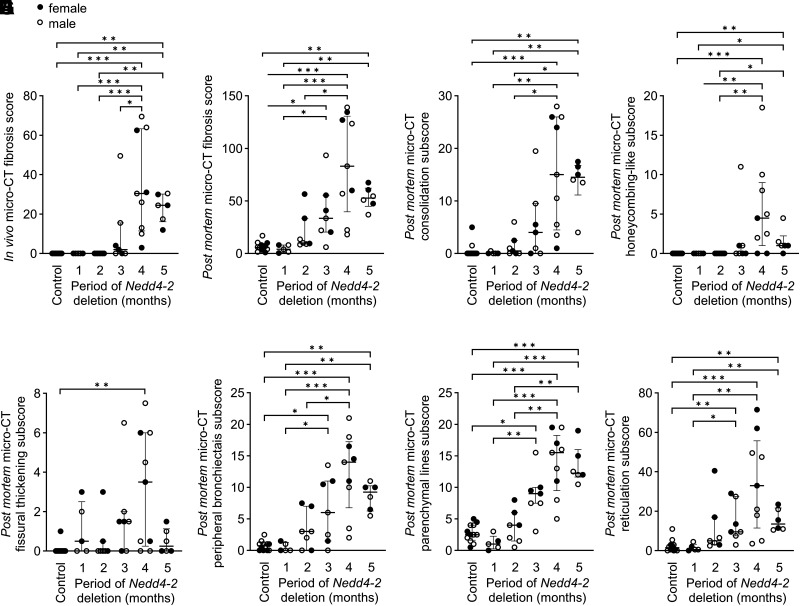
Development of radiological features of IPF-like lung disease in conditional *Nedd4-2^−/−^* mice. *A*: in vivo micro-CT fibrosis score along disease development after 1, 2, 3, 4, and 5 mo of doxycycline induction in conditional *Nedd4-2^−/−^* mice and controls. Post mortem micro-CT fibrosis score (*B*), consolidations (*C*), honeycombing-like lesions (*D*), fissural thickening (*E*), peripheral bronchiectasis (*F*), parenchymal lines (*G*), and reticulations (H) detected by high-resolution micro-CT in conditional *Nedd4-2^−/−^* mice and controls. Females are indicated as filled circles, males are indicated as open circles. *n* = 5–12 mice/group. FDR-adjusted *P* values are indicated as *P < 0.05, **P < 0.01, ***P < 0.001. IPF, idiopathic pulmonary fibrosis; micro-CT, microcomputed tomography.

The higher resolution of post mortem micro-CT allowed us to further characterize the onset and progression of fibrotic lesions and distinguish consolidations, honeycombing-like lesions, fissural thickening, peripheral bronchiectasis, parenchymal lines, and reticular structures in the cross-sectional study groups (Fig. 2*D* and Fig. 3, *B*–*H*). In these groups, there were no fibrotic changes detectable by in vivo micro-CT fibrosis scores in conditional *Nedd4-2^−/−^* mice after 1 and 2 mo of induction. However, in 4 and 5-mo-induced conditional *Nedd4-2^−/−^* mice in vivo micro-CT fibrosis scores were significantly elevated compared with control mice (Fig. 3*A*). After in vivo scans, we performed post mortem scans in these age- matched cross-sectional study cohorts. In general, high-resolution post mortem scans were able to detect even small changes in lung morphology, leading to higher post mortem fibrosis scores and a significant difference between conditional *Nedd4-2^−/−^* and control mice already after 3 mo of induction (Fig. 3*B*). The post mortem micro-CT fibrosis subscores revealed a similar dynamic change of consolidations as the in vivo micro-CT fibrosis scores (Fig. 3*C*). These analyses showed that peripheral bronchiectasis was already detectable after 2 mo in a subset of conditional *Nedd4-2^−/−^* mice and in all conditional *Nedd4-2^−/−^* mice after 4 and 5 mo of doxycycline induction (Fig. 3*F*). Parenchymal lines and reticulations showed a similar dynamic of which the latter accounted for the largest portion of the total fibrosis score across all age groups (Fig. 3, *B*, *G*, and *H*). Honeycombing-like lesions were mostly seen at later stages of the disease or in very sick mice and were not observed at 1 and 2 mo (Fig. 3*D*). The subscores for fissural thickening were variable along the age groups 1, 2, and 3 mo in conditional *Nedd4-2^−/−^* mice and significantly elevated at 4 mo in conditional *Nedd4-2^−/−^* mice compared with control (Fig. 3*E*).

### Micro-CT Fibrosis Score Correlates with Lung Function Impairment in Conditional *Nedd4-2*^−/−^ Mice

**Figure 4.**
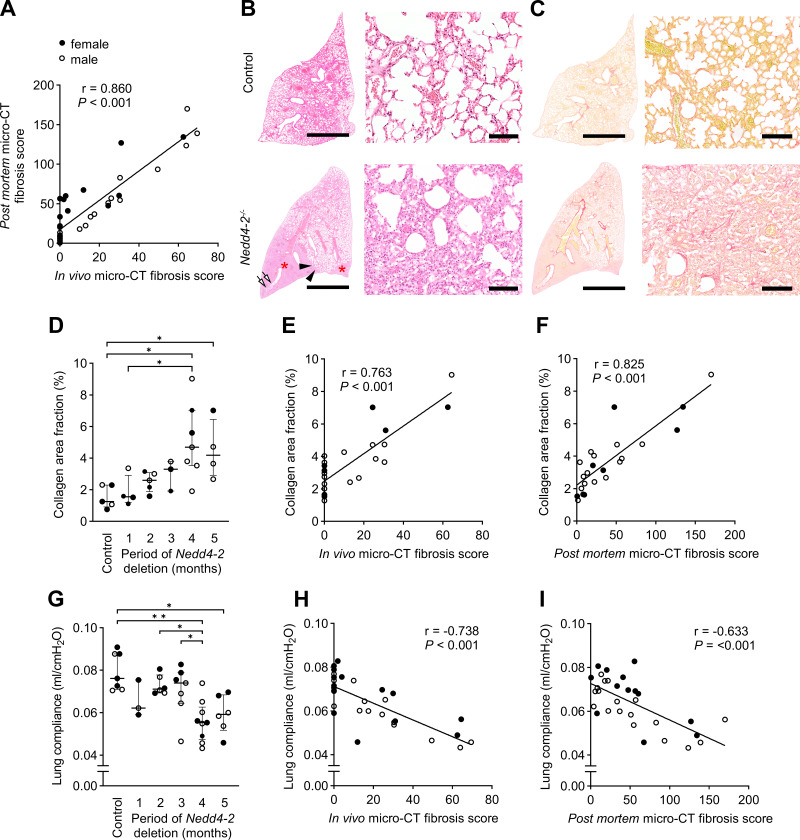
Micro-CT fibrosis score negatively correlates with impairment in pulmonary function in conditional *Nedd4-2^−/−^* mice. *A*: correlation between fibrosis scores determined by in vivo micro-CT and post mortem micro-CT. *n* = 31 mice. *B*: representative micrographs of H&E-stained lung sections of 4-mo-induced conditional *Nedd4-2^−/−^* and control mice. Low magnification (*left*, scale bar 2.5 mm). Consolidations (red asterisks), honeycombing-like lesions (black arrowheads), and peripheral bronchiectasis (white arrows). Higher magnification of cystic structural lung damages in the periphery of the lung (*bottom right* image, scale bar 100 µm). *C*: representative micrographs of histological sections stained for picrosirius red from 4-mo-induced conditional *Nedd4-2^−/−^* and control mice at low (*left*, scale bar 2.5 mm) and high magnifications (*right*, scale bar 100 µm). *D*: quantification of collagen in picrosirius red-stained lung sections showing the collagen area fraction in controls and conditional *Nedd4-2^−/−^* mice after the indicated periods of induction. *n* = 3–6 mice/group. Correlation between collagen area fraction and in vivo micro-CT fibrosis score (*E*) or post mortem micro-CT fibrosis score (*F*). *n* = 22 mice. *G*: summary of lung compliance at different time points in conditional *Nedd4-2^−/−^* mice compared with controls. *n* = 3–7 mice/group. Correlation between lung compliance determined by pulmonary function testing and in vivo micro-CT fibrosis score (*H*) (*n* = 35 mice) and post mortem micro-CT fibrosis score (*I*) (*n* = 37 mice) in conditional *Nedd4-2^−/−^* mice. Females are indicated as filled circles, males are indicated as open circles. *P < 0.05, **P < 0.01. Spearman rank order correlation coefficient (*r*), and *P* values are provided. H&E, hematoxylin and eosin; micro-CT, microcomputed tomography.

Although the in vivo micro-CT images did not enable the identification of specific fibrotic lesions, the fibrosis scores obtained with in vivo and post mortem micro-CT images from conditional *Nedd4-2*^−/−^ mice in the cross-sectional study groups showed a strong correlation with each other (*r* = 0.860, *P* < 0.001) (Fig. 4*A*). We further compared micro-CT images with histological images of conditional *Nedd4-2^−/−^* and control mice (Fig. 4*B*). Findings such as traction bronchiectasis were also present in the histological images of conditional *Nedd4-2^−/−^* mice (Fig. 2*B*). Furthermore, we found cystic destructions of the subpleural spaces in conditional *Nedd4-2^−/−^* mice, which corresponded to honeycombing-like lesions in the micro-CT (Fig. 4*B*). By picrosirius red staining, we found increased collagen deposition in the corresponding regions of consolidation in micro-CT (Fig. 4, *C* and *D*). The quantification of the collagen-stained area showed an increasing collagen area fraction overtime (Fig. 4*D*) and was strongly correlated with the in vivo (*r* = 0.763, *P* < 0.001) as well as the post mortem (*r* = 0.825, *P* < 0.001) micro-CT fibrosis score (Fig. 4, *E* and *F*). To test the relationship between the micro-CT fibrosis score and pulmonary function, we assessed lung compliance before the post mortem micro-CT. Pulmonary function testing demonstrated a significant decrease in lung compliance in conditional *Nedd4-2*^−/−^ mice after 4 and 5 mo of induction compared with control mice (Fig. 4*G*) and showed a strong inverse correlation with the fibrosis scores from conditional *Nedd4-2*^−/−^ mice obtained from in vivo (*r* = −0.738, *P* < 0.001) and post mortem micro-CT images (*r* = −0.633, *P* < 0.001) (Fig. 4, *H* and *I*).

### Micro-CT of End-Stage IPF-Like Lung Disease in Moribund Conditional *Nedd4-2*^−/−^ Mice

**Figure 5.**
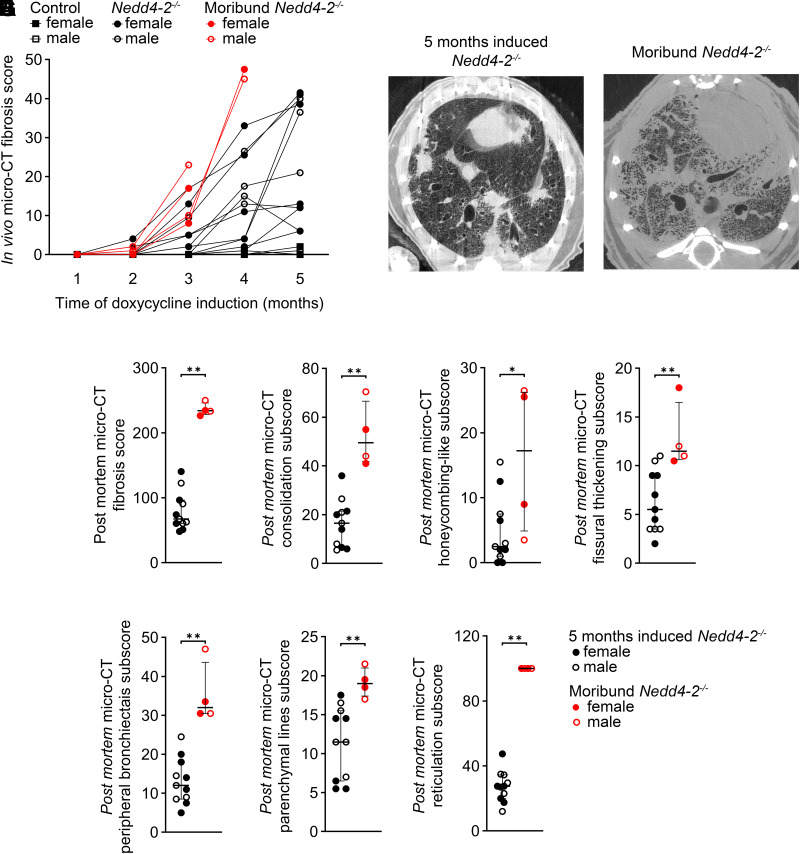
Micro-CT of end-stage IPF-like lung disease in moribund conditional *Nedd4-2^−/−^* mice. *A*: development of fibrosis scores in longitudinal micro-CT imaging studies of moribund conditional *Nedd4-2^−/−^* mice that had to be euthanized vs. conditional *Nedd4-2^−/−^* mice from the longitudinal study group that survived 5 mo of doxycycline induction. *B*: representative post mortem micro-CT images of a surviving conditional *Nedd4-2^−/−^* mouse and a moribund mouse that had to be euthanized after 4.5 mo. Summary of the post mortem micro-CT fibrosis scores (*C*), and the subscores for quantification of consolidation (*D*), honeycombing-like lesions (*E*), fissural thickening (*F*), peripheral bronchiectasis (*G*), parenchymal lines (*H*) and reticulations (*I*). *n* = 4–11 mice/group. *P < 0.05, **P < 0.01. IPF, idiopathic pulmonary fibrosis. micro-CT, microcomputed tomography.

During our study, four conditional *Nedd4-2^−/−^* mice from the longitudinal study group developed signs of severe respiratory distress with tachypnea and retractions associated with weight loss that had to be euthanized 3 to 4 mo after doxycycline induction (Fig. 5*A*). Post mortem micro-CT imaging of these moribund mice showed that IPF-like lung disease was substantially more severe in this group of mice with end-stage lung disease compared with conditional *Nedd4-2^−/−^* littermates from the longitudinal study cohort that survived and were studied 5 mo after doxycycline induction (Fig. 5). Specifically, reticulations and consolidations affected almost the entire lung (Fig. 5*B*), and the post mortem micro-CT fibrosis score and all of its subscores were markedly increased in these moribund mice compared with conditional *Nedd4-2^−/−^* mice in the longitudinal study group that were studied after 5 mo of induction (Fig. 5, *C*–*I*).

## DISCUSSION

In this study, we used micro-CT imaging to characterize the onset and spontaneous progression of IPF-like lung disease in conditional *Nedd4-2^−/−^* mice. With longitudinal in vivo micro-CT, we were able to closely monitor the development of fibrotic lesions from the onset to end-stage lung disease in moribund mice (Fig. 1 and Fig. 5). Higher resolution post mortem micro-CT imaging in age-matched mice provided more detailed insights into the specific structural changes and pattern of disease progression in this model of IPF-like lung disease (Figs. 2 and 3). Furthermore, we found that the fibrosis score determined from micro-CT imaging correlates with collagen deposition in the lung parenchyma and lung function impairment in conditional *Nedd4-2^−/−^* mice (Fig. 4). Collectively these data suggest that micro-CT imaging in conditional *Nedd4-2^−/−^* mice can be used for noninvasive testing of the in vivo effects of new therapeutic approaches or disease-relevant factors in both early and late IPF-like disease.

This longitudinal study extends our previous micro-CT imaging studies of advanced lung disease in conditional *Nedd4-2^−/−^* mice and provides important insights into the onset and progression of lung disease in this model of IPF (17). To date, there is little knowledge of the initial lesions of IPF and how they develop in vivo into the massive destruction of lung tissue that is observed in patients with IPF. We therefore used the conditional *Nedd4-2^−/−^* mice to obtain insights into the in vivo development of IPF-like lung disease (17). Similar to previous studies in conditional *Nedd4-2^−/−^* mice with advanced IPF-like lung disease, we found radiomorphological characteristics of consolidations, reticulations, traction bronchiectasis, and honeycombing-like lesions, resembling a UIP pattern in this mouse model (Figs. 2 and 3) (17). We found that fibrosis scoring based on in vivo and post mortem micro-CT shows a high correlation with each other (Fig. 4), indicating that the use of in vivo micro-CT imaging is useful to reliably assess disease progression and potentially response to therapy. However, in vivo micro-CT allowed only limited information about detailed structural characteristics of fibrotic changes due to motion artifacts caused by cardiac and respiratory motion, as well as a somewhat lower resolution of 35 µm. The subsequent *p* post mortem micro-CT with a resolution of 9 µm provided a more detailed characterization of the morphological changes in this model of IPF-like lung disease. Regarding the sequence of disease development, rather subtle and nonspecific changes, such as peripheral bronchiectasis or mild reticulations, are present in some mice already after 2 mo of deletion of conditional *Nedd4-2*^−/−^ and progress overtime. Honeycombing-like lesions can also be reliably distinguished by micro-CT with advanced IPF-like disease after 4 and 5 mo of doxycyline induction (Fig. 2, Fig. 3, and Fig. 5). This observation is consistent with our previous work, including CT imaging and histopathology studies at limited time points, in which only mild nonspecific histomorphological changes were detected in 2-mo-induced conditional *Nedd4-2*^−/−^ mice, such as mild macrophage-dominated inflammation associated with an increase in septal wall thickening, which could only be detected by studies of the ultrastructure of the lung (17, 39). At later time points, after 3 to 4 mo of induction, typical histological features of an IPF-like lung disease with honeycombing-like lesions and fibroblastic foci could be observed (17). After 4–5 mo of induction, we further observe significant deposition of collagen in the lung parenchyma together with a decline in pulmonary function. This suggests that at the beginning of the manifestation of IPF-like lung disease, rather nonspecific and subtle changes determine the morphology, and that the typical UIP pattern already reflects irreversible lung changes of advanced lung disease (Fig. 2, Fig. 3, and Fig. 5).

A potential limitation of this study is found in the correlation of the in vivo micro-CT fibrosis scores with the collagen proportionate area (Fig. 4*E*) and lung compliance (Fig. 4*H*), in which a considerable amount of data is clustered along the ordinate axis. This skewness of the data indicates a lower sensitivity of the in vivo micro-CT fibrosis score in detecting early, minor lung changes. This limitation should be considered when using the in vivo micro-CT score in longitudinal studies.

In our study, we observed a decrease in fibrosis scores after 5 mo of conditional *Nedd4-2* deletion in the cross-sectional cohorts in Fig. 3, after peaking at 4 mo. This could be misinterpreted as a possible spontaneous reversibility of the phenotype of our mouse model. An explanation for this observed trend in the cross-sectional study groups most likely is a survival bias, where mice with less disease activity are more likely to survive up to 5 mo, whereas mice with more severe disease manifestations do not survive as long. This would result in fibrosis scores being more pronounced at earlier time points. Furthermore, we also performed longitudinal micro-CT scans in another group of conditional *Nedd4-2*^−/−^ mice, as shown in Fig. 1 and Fig. 5. Since these longitudinal analyses showed a continuous progression of the disease overtime, a potential spontaneous reversibility of the disease in conditional *Nedd4-2*^−/−^ mice can even be excluded.

The comparison of micro-CT scores from mice that received single versus multiple in vivo micro-CT scans also allowed us to assess potential effects of radiation-induced lung injury, which is important since it may mimic IPF-like lung changes (40). We attempted to minimize radiation exposure by keeping the scanning time as short as possible and waiving respiratory-triggered examinations, as it would require an even higher radiation dose due to longer scan times. We observed increased fissural thickening and reticulation in conditional *Nedd4-2*^−/−^ mice subjected to repeated versus single micro-CT examinations (Supplemental Fig. S2). Although little is known about the long-term consequences of radiation on IPF, studies of patients with interstitial lung disease who have undergone radiation therapy for malignant lung tumors indicate that they are at higher risk for acute radiation-induced lung injury (41–43). Although the subscores of fissural thickening and reticulation showed a similar trend in control animals (Supplemental Fig. S2), our data suggest that the lung tissue of conditional *Nedd4-2*^−/−^ mice may be more susceptible to damaging events than it is the case in healthy lungs. However, the changes noted in the control mice may also be nonspecific postinflammatory changes, because the measured radiation dose of ∼0.39 Sv per scan for in vivo micro-CT is well below previously reported doses, which also did not result in significant fibrotic changes (40, 44). Importantly, our data demonstrate the feasibility of in vivo micro-CT imaging without significant radiation-induced lung injury. The strong correlation between collagen deposition as well as lung compliance with micro-CT fibrosis score reinforces the importance of this method in predicting the functional and histomorphological outcome in mice. In this context, micro-CT offers a distinct advantage over pulmonary function testing and histology, which are invasive in mice and helps to reduce the number of animals needed, in accordance with animal welfare and 3 R principles.

In our study, we found striking differences in the pattern and development of pulmonary fibrosis in conditional *Nedd4-2*^−/−^ mice compared with other experimental models of IPF. One of the most commonly used mouse model of BILF (9). Micro-CT studies in these mice showed that fibrotic alterations occurred rapidly after bleomycin instillation, occupying primarily the mid-to-upper regions of the lung with a dorsocentric distribution that emanates from bronchovascular areas to the periphery (24, 25, 45–47). We found that the IPF-like lung disease in conditional *Nedd4-2*^−/−^ mice develops spontaneously, progresses over several months in a chronic, initially slowly progressing way that rapidly worsens at the end of disease development. Further fibrotic lesions in these mice exhibit a basolateral distribution originating from the periphery of the lung, thus more closely mirroring the pattern observed in patients with IPF (48). Taken together, the conditional *Nedd4-2*^−/−^ mice are distinct from the mouse model of BILF demonstrated by a greater similarity to IPF in patients, which has previously also been shown at the histological level with microscopic honeycombing, fibroblast foci-like structures, bronchiolization of peripheral airways, and at the molecular level with altered pulmonary expression of Muc5b and a similarly altered proteomic signature as in IPF (17).

Our results also enable a more detailed comparison of radiological features of the development of lung disease in conditional *Nedd4-2*^−/−^ mice and patients with IPF. The radiological features of UIP, the hallmark of IPF, were detailed in the 2018 guidelines for the diagnosis of IPF (48) and revised in the new official ATS/ERS/JRS/ALAT clinical practice guideline, which opted to retain the four established classifications of CT patterns in IPF: “UIP pattern,” “probably UIP pattern,” “indeterminate for UIP,” “CT findings suggestive of an alternative diagnosis,” which are based on the distribution and features of CT findings (1). Moreover, apart from IPF and other relatively rare interstitial lung diseases (ILDs), incidental interstitial lung abnormalities (ILAs) are increasingly reported in cross-sectional imaging studies from smokers, for example in the setting of lung cancer screening (49). Since diagnosis is usually made late, early findings in IPF have not been defined, which also includes a lack of knowledge on actionable findings in the case of asymptomatic and incidentally found ILA at imaging (49, 50). The conditional *Nedd4-2*^−/−^ mice showed all CT features of a UIP pattern (honeycombing with or without traction bronchiectasis and presence of irregular thickening of interlobar septa). The distribution of morphological abnormalities in conditional *Nedd4-2*^−/−^ mice is predominantly subpleural and basal as well as mostly heterogeneous. However, conditional *Nedd4-2*^−/−^ mice also showed consolidations as a substantial feature, which are suggestive of alternative processes, such as consolidating pneumonia. This difference from human IPF could be related to anatomical differences between mice and humans. Since the airways in mice are much smaller, they might be inherently more susceptible to conditions such as atelectasis, which present as consolidations on CT. Notably, since previous work in this mouse model has shown overexpression of Muc5b, particularly in the periphery of the lung, and decreased mucociliary clearance due to increased transepithelial ENaC-mediated Na^+^ absorption leading to airway surface dehydration, the observed phenomenon could thus represent atelectasis due to impaired mucociliary clearance and/or mucus plugging (17, 51, 52). However, a predominant peribronchovascular pattern with subpleural sparing suggestive for NSIP was not found. Collectively, the micro-CT findings from our study, which highlight predominant UIP features, combined with our previous observations of IPF-signature lesions in histology and shared molecular signatures with IPF patients, support the relevance of conditional *Nedd4-2*^−/−^ mice as a model of IPF, and potentially other ILD and ILA.

Given the importance of sex as a variable in biomedical research, subgroup analyses were performed in our study to investigate potential sex-specific differences in the progression of IPF-like lung disease in conditional *Nedd4-2*^−/−^ mice. In all investigated groups we found no significant differences in fibrosis scores between the sexes in our study. However, these conclusions are based on a relatively small sample size, which limits the statistical power and generalizability of our results.

In this study, we used an adapted micro-CT-based fibrosis score for quantifying fibrotic lesions in a standardized manner (24, 25). The decision to use a semiquantitative visual score was driven by its simplicity and its reliance on established radiological CT variables (52). However, scoring systems such as this carry the risk of information loss, as they only capture features that meet predefined criteria. In a longitudinal study in which most diagnostic criteria were derived from advanced disease stages with irreversible changes, there is a possibility that precursor lesions may not be adequately represented in the scoring system. This could result in missing crucial clinical insights into disease evolution. Although recent advancements have produced computer tools that promise greater precision in detecting lung damage, they also have potential drawbacks, particularly in pulmonary fibrosis, since they are more susceptible to misinterpretation of shrinkage and to noise and motion artifacts. Although semiquantitative scores are widely accepted in clinical settings, automated reading might offer advancements in the future, though with its own set of challenges.

In summary, we demonstrate the progressive nature of pulmonary fibrosis in conditional *Nedd4-2*^−/−^ mice by longitudinal in vivo micro-CT. Furthermore, post mortem micro-CT provides detailed descriptions of structural lung damage at various stages, a novel contribution to the field. Beyond tracking fibrotic progression, our approach integrates longitudinal in vivo micro-CT with high-resolution post mortem imaging and lung function tests, offering a nuanced view of the correlation between radiological and physiological changes. We also introduce and validate a refined micro-CT scoring system tailored to this model’s pathology, enhancing the accuracy of our findings. Finally, our research illuminates the early stages of IPF-like disease progression, setting the stage for future therapeutic strategy development and evaluation.

### Perspectives and Significance

Collectively, longitudinal micro-CT imaging facilitates noninvasive assessment of pulmonary fibrosis of the entire lung in vivo and serves as a valuable quantitative tool for future studies, especially to elucidate disease mechanisms and risk factors of disease progression or for preclinical evaluation of novel therapeutic strategies.

## DATA AVAILABILITY

The data presented in this study are available on request from the corresponding author.

## SUPPLEMENTAL MATERIAL

10.6084/m9.figshare.24061650 Supplemental Table S1: https://doi.org/10.6084/m9.figshare.24061650.

10.6084/m9.figshare.25669332 Supplemental Table S2: https://doi.org/10.6084/m9.figshare.25669332.

10.6084/m9.figshare.24061662 Supplemental Fig. S1: https://doi.org/10.6084/m9.figshare.24061662.

10.6084/m9.figshare.25669299 Supplemental Fig. S2: https://doi.org/10.6084/m9.figshare.25669299.

10.6084/m9.figshare.25669305 Supplemental Fig. S3: https://doi.org/10.6084/m9.figshare.25669305.

## GRANTS

This study was supported by grants from the German Federal Ministry of Education and Research (82DZL00401, 82DZL004A1, 82DZL009B1) and the German Research Foundation (CRC 1449—project 431232613; subproject Z02). Funders had no involvement in the collection, analysis, interpretation of data, in the writing of the report, and in the decision to submit the article for publication.

## DISCLOSURES

No conflicts of interest, financial or otherwise, are declared by the authors.

## AUTHOR CONTRIBUTIONS

D.H.W.L., P.K., W.L.W., M.M., C.B., T.S., W.S., H.-U.K., M.A.M., J.D., and M.O.W. conceived and designed research; D.H.W.L., P.K., W.L.W., M.M., C.B., T.S., W.S., H.-U.K., M.A.M., J.D., and M.O.W. performed experiments; D.H.W.L., P.K., W.L.W., M.M., C.B., T.S., W.S., H.-U.K., M.A.M., J.D., and M.O.W. analyzed data; D.H.W.L., P.K., W.L.W., M.M., C.B., T.S., W.S., H.-U.K., M.A.M., J.D., and M.O.W. interpreted results of experiments; D.H.W.L., P.K., W.L.W., M.M., C.B., T.S., W.S., H.-U.K., M.A.M., J.D., and M.O.W. prepared figures; D.H.W.L., P.K., W.L.W., M.M., C.B., T.S., W.S., H.-U.K., M.A.M., J.D., and M.O.W. drafted manuscript; D.H.W.L., R.E.M., and C.D. edited and revised manuscript; D.H.W.L., P.K., W.L.W., M.M., C.B., T.S., R.E.M., C.D., W.S., H.-U.K., M.A.M., J.D., and M.O.W. approved final version of manuscript.
